# Extended Endonasal Endoscopic (EEE) Surgery with Almost No Use of Adjuvant Radiotherapy for Juvenile Nasopharyngeal Angiofibroma (JNA)

**DOI:** 10.3390/medicina59091620

**Published:** 2023-09-07

**Authors:** Shamsul Alam, Bipin Chaurasia, Mohsin Ali Farazi, Gianluca Ferini, Abu Saleh Mohammad Abu Obaida, Atiqul Islam, Abu Naim Wakil Uddin, Asifur Rahman

**Affiliations:** 1 Department of Neurosurgery, Bangabandhu Sheikh Mujib Medical University, Dhaka 1000, Bangladeshwakilan@yahoo.com (A.N.W.U.); bijoun14@yahoo.com (A.R.); 2 Department of Neurosurgery, Neurosurgery Clinic, Birgunj 44300, Nepal; 3 Department of Neurosurgery, Khulna Medical College, Khulna 9100, Bangladesh; 4 Radiation Oncology Unit, Department of Radiotherapy, REM Radioterapia Srl, 95029 Viagrande, Italy; 5 Department of Neurosurgery, National Institute of Neuroscience, Dhaka 1207, Bangladesh; kazidmc@gmail.com

**Keywords:** juvenile nasopharyngeal angiofibroma (JNA), extended endonasal endoscopic (EEE) approach, staging of JNA, Denker’s anteromedial maxillotomy

## Abstract

*Background and Objectives*: Juvenile nasopharyngeal angiofibroma (JNA) is an angiomatous hamartoma of the nasal cavity. It is a benign but locally aggressive vascular tumor of the nasopharynx affecting adolescent males. Many surgical procedures are in practice, but the extended endonasal endoscopic (EEE) approach for JNAs is a suitable and effective technique. *Materials and Methods*: Fifteen adolescent patients having JNA who underwent extended endonasal endoscopic (EEE) surgery from January 2010 to January 2022 were studied retrospectively. Patients having residual and recurrent JNAs and those who underwent surgery other than EEE were excluded. *Results*: The average age of the patients was 18.3 years of age. A total of six patients (40%) each had stage V and IV while three patients (20%) had stage III JNAs. Gross total removal was achieved in eight (53.3%) patients and seven (43.7%) had partial removal. There was no per or postoperative mortality. All the patients had at least 3 years of postoperative follow-up and during follow-ups, seven patients were found to have residual tumors, and two had recurrences. *Discussion*: During the last decades, the endoscopic approach for the resection of JNAs has gained increasing popularity due to its obvious advantages over transfacial approaches. The magnified and angled field of view “behind the corner” helping in a more complete inspection for the resection and shorter hospitalization time makes it a better choice than the other approaches. *Conclusions*: Endoscopy is an excellent approach for primary JNA. It allows well visualization and precise removal of the angiofibroma. An endoscopic multiangle, multicorridor skull base approach including Denker’s anteromedial maxillotomy is suitable and preferable for the resection of extensive JNAs.

## 1. Introduction

Juvenile nasopharyngeal angiofibroma (JNA) is an uncommon angiomatous hamartoma of the nasal cavity. It is a benign but locally aggressive vascular tumor of the nasopharynx and accounts for only 0.05% to 0.5% of head and neck tumors with a high incidence of recurrence [1,2,3]. JNA, as the nomenclature advocates, affects almost exclusively adolescent males and the incidence is 1:150,000. As occurrences in young adults from 14 to 25 years have been reported in the literature, the terminology “juvenile” exclusively for JNA is deemed to be a misnomer. Females can also be rarely affected [2,4,5].

JNAs are thought to originate from the pterygopalatine fossa around the aperture of the pterygoid (vidian) canal. And from its origin in the vicinity of the superior margin of the sphenopalatine foramen, they grow into other surrounding areas in multiple directions, particularly into the nasal and paranasal cavities and the skull base with infrequent orbital and intracranial extensions in advanced stages. The pattern of invasion into local structures actually is unpredictable and often atypical. JNAs usually present with unilateral or bilateral nasal block with repeated and profuse epistaxis. Advanced stages of JNAs may lead to facial dysmorphism as well as conductive type of hearing loss due to eustachian tube block. Erosion of the superior orbital fissure involving the third and sixth nerves may produce diplopia. And intraorbital extension may produce proptosis [4,6,7].

Genetic studies have demonstrated a close relation between JNA and androgen receptor expression, signifying JNAs to be androgen-dependent, which in turn explains the male predominance of this set of particular tumors [4,8]. Another gene, the GSTM 1 gene, has also been implicated in the development of JNA where loss of expression of the GSTM 1 is found in these lesions [9].

Patients usually come for management at a late stage of the disease with the classical symptoms of severe epistaxis and progressive nasal obstruction resulting from a nasopharyngeal mass [2]. Computed tomography (CT) scan is the gold standard for the diagnosis of JNA. Magnetic resonance imaging (MRI) of the paranasal sinus (PNS) also gives valuable information regarding the extent of involvement along with skull base infiltration. Antral sign or Holman–Miller sign (forward bowing of posterior wall of maxilla) is pathognomic of angiofibroma. Digital subtraction angiography (DSA) of the carotid arteries is helpful to see the extension of growths and feeding vessels [2,6,7,10].

Standard therapeutic options for JNAs include surgery, open or endoscopic or combined, and/or definitive radiotherapy. Other less common options are percutaneous sclerotherapy, chemotherapy, hormonal therapy, cryosurgery or sometimes gamma knife radiosurgery of residual tumors. However, surgery and/ or RT remain to be the most effective modalities of treatment. Nowadays, surgery by extended endonasal endoscopic (EEE) approach has gained popularity for its ease and efficacy during the intraoperative and postoperative periods rather than open surgery [1,2,3].

## 2. Methods

In this retrospective study, 15 adolescent patients having JNA were analyzed from January 2010 to January 2022. All the patients underwent extended endonasal endoscopic (EEE) surgery. Only the newly diagnosed cases and who had at least 3 years of postoperative follow-up were included in this study. Patients having residual and recurrent JNAs and those who underwent surgery other than EEE surgery like those with Weber–Ferguson incision, which was commonly practiced initially, were excluded from this study. Patients’ follow-up was performed by nasal endoscope on 7th day then 1 month and ultimately 3 months after surgery. A postoperative CT scan was conducted within the first week of the operation and an MRI of the paranasal sinuses was conducted 3 months following surgery, then MRI was performed every 6 months up to 3 years.

## 3. Operative Procedure

The two-surgeon, four-hand technique endonasal endoscopic approach, which improves hemostasis, maximizes surgical access, and minimizes operative time, was used in all the cases. Zero degree (0°) 4 mm endoscope and 18 cm long telescope were used in all cases. A central venous (CV) line was instituted through the right subclavian vein and two to three venous accesses for blood replacement were kept ready. Six to eight units of whole blood were arranged and kept ready for perioperative use. As there is always a risk of massive blood loss, several precautions such as rapid induction, hypotensive anesthesia, and hypothermia have been taken as recommended in the literature to lessen bleeding. Furthermore, the patient was placed in the reverse Trendelenburg position for the reduction in blood flow to the tumor site with the aid of gravity.

## 4. Technical Notes

Irrigation of the nose with normal saline and diluted povidone iodine was performed and adrenaline-soaked gauze pack or Merocel pack was applied to aid hemostasis of the tumor surface in the nasal cavity. The part of the tumor in the nasal cavity, when present, was removed with Blakesley forceps. Inferior turbinectomy and septectomy were performed to facilitate the four-handed technique and to visualize the whole nasopharyngeal part of the tumor.

The anterolateral wall of the maxilla was exposed with Denker’s procedure and drilled up to the zygomatic eminence laterally, canine eminence inferiorly, and infraorbital nerve superiorly, taking care not to injure it. The lateral wall of the nose which is the medial wall of the maxilla was exposed and drilled up to the level of the nasolacrimal duct which was exposed and transected. Posteriorly, bone was drilled to the level of the junction of the palatine bone and the medial pterygoid plate, where the descending palatine neurovascular bundle was seen and cauterized. The posterior wall was drilled towards the meeting point with the anterolateral wall to reach the infratemporal fossa. The tumor was manipulated medially to expose, identify, and cauterize the internal maxillary artery. The parts of the tumor in the infratemporal fossa and the cheek were removed by dissection and gentle traction with curved Luc’s forceps. In the pterygoid wedge, the tumor was made free by resecting the attachments around and was removed in piecemeal or en plaque. The rest of the tumor in the pterygoid wedge was removed by drilling the wedge. The part of the tumor extending into the quadrangular space was removed by drilling the anterolateral wall of sphenoid sinus. The medial wall of the paraclival ICA was skeletonized and transpositioned with doppler assistance to remove the remainder of the tumor in the quadrangular space. Following transpositioning the ICA, removing the parts of the tumor from both sides of the cavernous sinus was possible, saving the cranial nerves on the lateral wall.

## 5. Results

**Figure 1:**
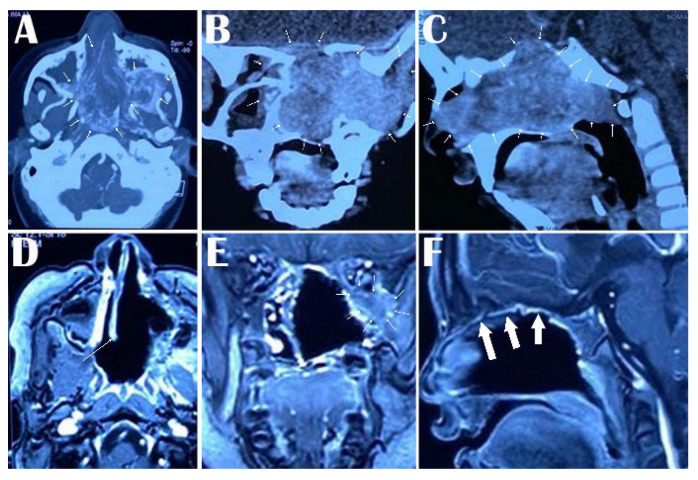
Preoperative contrast-enhanced CT scan of the head showing extent of JNA with bone erosion in all the sections in the nasal cavity, nasopharynx, and infratemporal fossa in axial section arrows around the lesion in (**A**), nasal cavity, pterygopalatine fossa, inferior orbital fissure and infratemporal fossa in coronal section (arrows around the lesion in (**B**)) and nasal cavity, ethmoid sinus, sphenoid sinus, and nasopharynx in sagittal section (arrows around the lesion in (**C**)). Postoperative Gd-enhanced MRI showing the extent of removal of the JNA from the nasal cavity, nasopharynx, and infratemporal fossa in the axial section (arrow showing the nasal septum in (**D**)), complete removal from the nasal cavity, infratemporal fossa except a small residue in the infraorbital fossa in coronal section (arrows around the residual part in (**E**)) and total clearance from the nasal cavity, ethmoid sinus, sphenoid sinus, and nasopharynx in sagittal section (arrows showing the thickened nasal mucosa in (**F**)).

**Figure 2:**
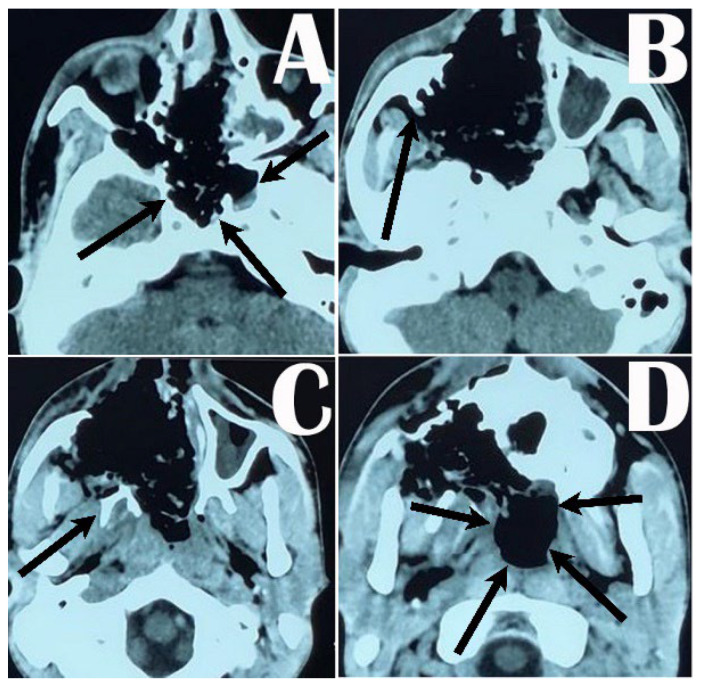
Postoperative CT scan of the head showing extent of tumor removal from the nose, oropharynx, sphenoid sinus, and clival fossa (arrows around the clival fossa in (**A**)); Denker’s maxillotomy and removal of the tumor from the maxilla and pterygopalatine fossa (arrow showing the part of the posterior wall of maxilla in (**B**) and pterygoid process in (**C**)), and removal of the tumor from the nasopharynx and submaxillary area (arrows around the nasopharynx (**D**)).

**Figure 3:**
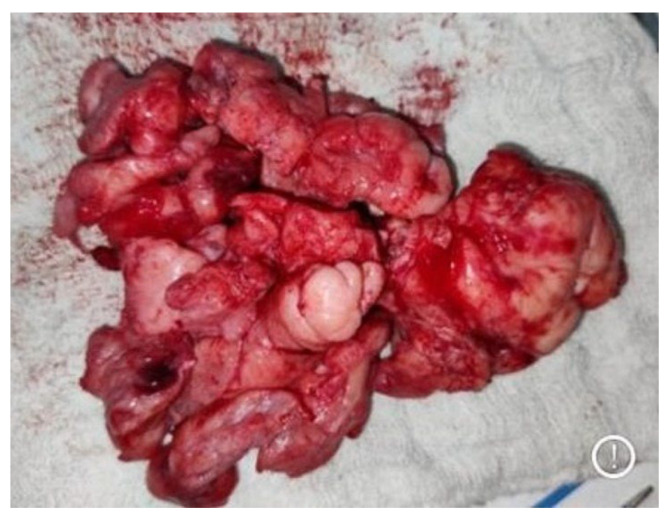
Bulk of excised multilobulated angiofibroma.

The average age of the patients was 18.3 years of age (Table 1). The most common grading of angiofibroma in our patients was stage V (40%) and stage IV (40%) (six cases each). Stage III (20%) was the least common having three (20%) cases. Most of the patients presented with epistaxis, progressive nasal obstruction, and facial deformity. Commonly involved structures were the nasopharynx, sphenopalatine fossa and maxillary fossa, and right nasal cavity (Table 1). The cavernous sinus was involved in seven cases (46.7%). Peroperative blood loss on average was 1500 mL. Peroperative hypotension that led to cardiac arrest developed in two cases that had to be managed by blood transfusion and CPR and/or abandoning of the surgical procedure. Gross total removal was achieved in eight (53.3%) patients while subtotal removal was performed in seven (43.7%) patients (Table 1 and Figure 1, Figure 2 and Figure 3). No patient died during surgery or within the 3 years postoperative follow-up period. All seven patients that had subtotal removal had residual tumor in their follow-up scans. Five patients underwent re-exploration within 6 months of the first surgery without any RT as the follow-up scans showed some increase in their size. Two patients were re-explored through the endonasal endoscopic approach and three underwent transcranial re-exploration through fronto-parietal-orbito-zygomatic (FTOZ) approach and the other two were kept under observation as the residual tumors were small and remained static in size. Two of the patients having gross total removal showed recurrence of tumor within 2 to 2½ years following surgery. Both patients were re-explored and gross total removal was achieved again. Before the re-exploration of one patient, radiotherapy was recommended before endoscopic surgery, and following radiotherapy, tumor was found to be firm and less vascular. None of the patients were advised to undergo radiotherapy following gross total removal at the first surgery. Epistaxis and facial swelling were the most common complications following surgery while two patients developed nasal deformity. Seven patients had no complications (Table 1).

## 6. Discussion

Juvenile nasopharyngeal angiofibroma is histologically benign but is a locally aggressive and very vascular head and neck tumor that may involve the skull base and even extend intracranially. JNAs are circumscribed, lobulated, non-capsulated, mucosa-covered masses that spread submucosally and tend to extend along natural foramina and fissures. Although JNAs do not invade; nevertheless, they frequently erode by pressure atrophy. Drenched by rich multiple anastomotic vascular supplies from the maxillary artery, ascending pharyngeal artery, ipsilateral internal carotid artery, or the contralateral external carotid artery, JNAs may result in significant blood loss following minor trauma or biopsy or often may bleed spontaneously. Other than the common presentation of nasal block and epistaxis, larger tumors tend to cause facial deformity. Proptosis and diplopia may result from the involvement of the orbit. Cranial nerve involvement may lead to facial hypoesthesia and visual loss [1,2,4,11].

Open surgery or endoscopic surgery with adjuvant radiotherapy (RT) or definitive radiotherapy alone are the usual standard therapeutic options. Percutaneous sclerotherapy, chemotherapy, hormonal therapy, cryosurgery or sometimes gamma knife radiosurgery are also in practice. Surgery remains the gold standard of treatment often complemented by RT as treatment modality when efficacy is concerned [1,2,11].

Different surgical approaches are practiced which include endonasal endoscopic approach with or without vascular embolization, endoscopic approach with or without added labiogingival incision, and open approaches like LeFort osteotomies, transoral, transfacial, transpalatal, or transmaxillary approaches, midfacial degloving, lateral rhinotomy, ITF and middle fossa approaches, or craniofacial approach [1,11,12,13,14].

Depending on the extension of the tumor and its spreading into and involving the surrounding structures, several staging criteria have been set and practiced. Although many staging systems are there, the staging systems of Fisch and Radkowski have been the most used and popular ones [15,16]. However, many modifications have been made for convenience and all have some utility in staging JNAs [17,18,19,20,21]. In 2010, the University of Pittsburgh Medical Center (UPMC) staging system for JNA was introduced, emphasizing intracranial extension, and the extent of vascular supply from the internal carotid artery that is particularly apt for endoscopic endonasal surgical approaches [22]. All these staging systems are convenient for surgical planning, assessment of need for adjuvant therapy, and evaluating the possibility of residue and recurrence. We followed the UPMC staging system for assessment and planning of management in our series.

Most of the patients in most of the series have been shown to present at variable stages of the disease, mostly varying between IIC and IIIB in different staging systems, and a few at stages IV and V. However, tumors extending into the skull base, middle fossa, and orbit necessitate wide exposure. Wide exposure carries the risk of increasing operative morbidity and complications that might prevent a safe and complete tumor removal on some occasions. Defining the precise site and extension of tumor invasion is important for surgical planning and failing to identify the anatomical site may also lead to incomplete resections [4,12,13,14]. Owing to late presentation, most of the patients of JNA in our series came to us when they were already in stage III or more, by means of whatever staging system was used.

Over the last few decades, among surgical techniques, the endoscopic approach for the resection of JNA has gained increasing popularity due to its obvious advantages over external trans-facial approaches. The endoscopic approach has the advantage of avoiding facial incisions, osteotomies, and bone plating, which saves young patients from the risk of morbid craniofacial disfigurements. In addition, the magnified field of view and angled view “behind the corner” assist in complete inspection for the resection, aiding in shorter hospitalization time. This also lowers postoperative morbidity and preservation of both anatomy and physiology of the nose and surrounding areas. Earlier reports on the exclusive use of the endoscopic approach were restricted only to early-stage JNAs, which were confined to the nasal cavity, nasopharynx, ethmoid, and sphenoid sinuses with limited pterigo palatine fossa (PPF) extension [1,16,23,24,25]. Later on, some experienced centers successfully expanded the indications for EEE to more advanced lesions involving the infratemporal fossa (ITF) and parasellar area as well. Preoperative embolization, 24–48 h prior to surgery, may ease surgery by reducing intraoperative blood loss which would lead to less morbidity, more possibility of complete resection, and less recurrence [1,4,11]. Compared to other series, our series was relatively a smaller one, comprising only 15 patients. However, unlike other series, particularly of note is that most of our patients (80%) came with stage IV and V disease, whereas most of the patients in the other series presented with a maximum of IIIB stage with a few stage IV or V lesions. This is presumably due to the late presentation of the patients to us. Illiteracy, negligence about health problems, and sometimes financial constraints make the patients come to us very late when the disease has progressed very far.

Perioperative blood loss ranged in the literature from 342.3 mL to 2500 mL considering all approaches of open, combined, and purely endoscopic ones, which may increase up to 4000 mL in cases where the tumors extend to the skull base. Preoperative tumor embolization is an effective measure to reduce intraoperative blood loss. However, embolized or not, endoscopic surgery has less blood loss than open surgery [1,11,14]. In our series, peroperative blood loss was about 1500 mL on average. The increase in blood loss more than most of the series presumably occurred because of not embolizing any of the tumors preoperatively where excess blood loss even led to perioperative cardiac arrest in two of our patients. Another reason for the excess blood loss can be the bigger size with an extensive spread of the tumors in our series compared to other series.

The most common perioperative complication is hemorrhage while the common postoperative complications include CSF leak, trigeminal nerve, mostly V2 and V3 anesthesia, trigeminal neuralgia, transient abducens nerve palsy, palatal insufficiency, orbital hematomas, optic neuropathy, epiphora, trismus, and sinusitis. However, compared to open surgery, endoscopic surgery tends to have fewer number of complications [1,11,13]. During the surgery, the most common complication was hemorrhage in our cases as none of the patients had preoperative embolization of the tumors. Intraoperative blood loss can sometimes be life-threatening as we faced in two of our patients. Other than epistaxis and facial deformity, two of our patients also developed nasal deformity postoperatively. There was no postoperative CSF leak.

Although radical surgical resection is the treatment of choice, often, due to many unavoidable circumstances, some parts of the tumor had to be left behind. Different series describe detection of residual tumor in postoperative scans ranging between 8.7% to 33.3%. For residual tumors, different options are available, such as re-exploration, radiotherapy, or observation with serial follow-up imaging [11,14]. Nevertheless, the rate of residual tumor in our study was much higher than in the other studies. The higher number of patients with residual tumors, 46.7% patients in our study, was due to the unavailability of modern facilities in our setup during the earlier period of study, such as flow-seal for hemostasis, DSA set-up for preoperative vascularity assessment, and perioperative unilateral or bilateral ligation of the external carotid arteries. In a few cases, the surgery had to be abandoned for fear of losing the patient on the table from blood loss. Most patients with residual tumor were from the earlier period of the study when the surgical skill was in the developing phase. With the developing learning curve with time, the rate of excision has increased significantly and total removal has been possible in more patients from the later part of the series.

The rate of recurrence varies between 8.3% to 31.4% in different series. If recurrence occurs, various treatment modalities can be instituted including reoperation or watchful waiting. Some tumors may regress or stabilize when patients reach adulthood [12,14]. Only two (18.2%) of our patients showed recurrence in 3 years of follow-up which is consistent with other series.

Neither the postoperative residue nor the recurrence of the tumor were significantly different between any surgical approaches in any series.

## 7. Limitations

One major limitation of this study is the short follow-up period which is only 3 years. It would have been better if we could conduct a follow-up of the patients for at least for 5 years. However, keeping track of the patients for long follow-ups is difficult in our situation owing to the lack of proper data recording and financial constraints with the lack of health insurance policies. 

## 8. Conclusions

A complete clearance of the JNAs gives the advantage of avoidance of re-exploration and/or adjuvant radiotherapy and can reduce overall morbidity. Extended endonasal endoscopic (EEE) is an excellent safe and effective approach to achieve complete resection which can be modified as a multi-corridor skull base approach with synchronization of Denker’s anteromedial maxillotomy according to need. Nevertheless, for residual multicompartmental lesions with intracranial extension, transcranial re-exploration through fronto-parietal-orbito-zygomatic (FTOZ) approach can be a good option.

## Figures and Tables

**Table 1:** Showing gender, age, CT findings, approach, the extent of resection, and complications.

Case No, Gender & Age	Grading of Angiofibroma	C.T Finding	Name of Approach	Extent of Resection	Complication
Case 1 M/17 Y	Stage V	Multilobulated mass occupying the nasopharynx, sphenopalatine fossa, maxillary fossa, right nasal cavity	endoscopic, endonasal, trans maxillary pterygoid approach and middle fossa approach	partial removal	Facial swelling
Case 2 M/26 Y	Stage IV	Multilobulated mass occupying the nasopharynx, sphenopalatine fossa, maxillary fossa, right nasal cavity with superior orbital fissure and cavernous sinus	endoscopic, endonasal, trans maxillary pterygoid approach and middle fossa approach	partial removal	Epistaxis
Case 3 M/18 Y	Stage V	Multilobulated mass occupying the nasopharynx, sphenopalatine fossa, maxillary fossa, left nasal cavity	endoscopic, endonasal, trans maxillary pterygoid approach and middle fossa approach	partial removal	Epistaxis, facial swelling
Case 4 M/16 Y	Stage IV	Multilobulated mass occupying the nasopharynx, sphenopalatine fossa, maxillary fossa, right nasal cavity with superior orbital fissure and cavernous sinus	endoscopic, endonasal, trans maxillary pterygoid approach and middle fossa approach	total removal	Epistaxis, nasal deformity
Case 5 M/17 Y	Stage V	Multilobulated mass occupying the nasopharynx, sphenopalatine fossa, maxillary fossa, right nasal cavity with superior orbital fissure and cavernous sinus	endoscopic, endonasal, trans maxillary pterygoid approach and middle fossa approach	partial removal	No complication
Case 6 M/17 Y	Stage III	Multilobulated mass occupying the nasopharynx, sphenopalatine fossa, maxillary fossa, left nasal cavity	endoscopic, endonasal, trans maxillary pterygoid approach	partial removal	No complication
Case 7 M/17 Y	Stage V	Multilobulated mass occupying the nasopharynx, sphenopalatine fossa, maxillary fossa, right nasal cavity with superior orbital fissure and cavernous sinus	endoscopic, endonasal, trans maxillary pterygoid approach and middle fossa approach	total removal	Epistaxis, nasal deformity
Case 8 M/24 Y	Stage IV	Multilobulated mass occupying the nasopharynx, sphenopalatine fossa, maxillary fossa, right nasal cavity	endoscopic, endonasal, trans maxillary pterygoid approach and middle fossa approach	total removal	Epistaxis
Case 9 M/17 Y	Stage III	Multilobulated mass occupying the nasopharynx, sphenopalatine fossa, maxillary fossa, left nasal cavity	endoscopic, endonasal, trans maxillary pterygoid approach	total removal	Epistaxis, facial swelling
Case 10 M/16 Y	Stage IV	Multilobulated mass occupying the nasopharynx, sphenopalatine fossa, maxillary fossa, right nasal cavity with superior orbital fissure and cavernous sinus	endoscopic, endonasal, trans maxillary pterygoid approach and middle fossa approach	partial removal	No complication
Case 11 M/21 Y	Stage V	Multilobulated mass occupying the nasopharynx, sphenopalatine fossa, maxillary fossa, right nasal cavity	endoscopic, endonasal, trans maxillary pterygoid approach and middle fossa approach	total removal	No complication
Case 12 M/18 Y	Stage IV	Multilobulated mass occupying the nasopharynx, sphenopalatine fossa, maxillary fossa, left nasal cavity	endoscopic, endonasal, trans maxillary pterygoid approach and middle fossa approach	partial removal	Facial swelling
Case 13 M/18 Y	Stage III	Multilobulated mass occupying the nasopharynx, sphenopalatine fossa, maxillary fossa, right nasal cavity with superior orbital fissure and cavernous sinus	endoscopic, endonasal, trans maxillary pterygoid approach	total removal	No complication
Case 14 M/16 Y	Stage IV	Multilobulated mass occupying the nasopharynx, sphenopalatine fossa, maxillary fossa, right nasal cavity	endoscopic, endonasal, trans maxillary pterygoid approach and middle fossa approach	total removal	No complication
Case 15 M/17 Y	Stage V	Multilobulated mass occupying the nasopharynx, sphenopalatine fossa, maxillary fossa, right nasal cavity with superior orbital fissure and cavernous sinus	endoscopic, endonasal, trans maxillary pterygoid approach and middle fossa approach	total removal	No complication

## Data Availability

The data presented in this study are available in Table 1 of the article.
